# Supplementary material for: The Solvent Dimethyl Sulfoxide Affects Physiology, Transcriptome and Secondary Metabolism of Aspergillus flavus
Source: J Fungi (Basel). 2021 Dec 9;7(12):1055. doi: 10.3390/jof7121055 (PMC8703953; doi:10.3390/jof7121055)
Supplement: Supplementary file 1 [file jof-07-01055-s001.zip › JoF Costes et al Supp. Materials.pdf]

Journal of Fungi

Supplementary Materials

The solvent Dimethyl Sulfoxide affects physiology, transcriptome and secondary metabolism of *Aspergillus flavus*

Laura H. Costes[1], Yannick Lippi[1], Claire Naylies[1], Emilien L. Jamin[1,2], Clémence Genthon[3], Sylviane Bailly[1], Isabelle P. Oswald[1], Jean-Denis Bailly[1]* Olivier Puel[1]

[1] Toxalim (Research Centre in Food Toxicology), Université de Toulouse, INRAE, ENVT, INP-Purpan, UPS, Toulouse, laura.hm.costes@gmail.com (L.H.C.); yannick.lippi@inrae.fr (Y.L.); claire.naylies@inrae.fr (C.N.); emilien.jamin@inrae.fr (E.L.J); sylvianebailly7@gmail.com (S.B.) isabelle.oswald@inrae.fr (I.P.O.); jean-denis.bailly@envt.fr (J-D.B.); olivier.puel@inrae.fr (O.P.)

[2]Metatoul-AXIOM platform, MetaboHUB, National Infrastructure for Metabolomics and Fluxomics, Toulouse, France.

[3] INRAE, GeT-PlaGe, Genotoul, 31326, Castanet-Tolosan, France. clemence.genthon@inrae.fr (C.G.)

* Correspondence: jean-denis.bailly@envt.fr

Table of contents

Supplementary Figure S1. Survival rate of pigmented (control) and non-pigmented (D2) conidia related to the duration of mechanical disruption by ceramic beads.. 2

Supplementary Figure S2. A. LC-MS chromatograms of non-treated *Aspergillus flavus* culture extracts and Ustiloxin B authentic standard (ESI+ mode), *m/z* corresponding in positive mode to ustiloxin B is 646.32. B. Comparison of positive mass fragmentation (MS/MS) of m/z 646.32 between the non-treated *A. flavus* culture extract and ustiloxin B standard.............................. 3

Supplementary Figure S3. Fold Change expression of genes belonging to the mixed regulated leporins (#23), ditryptophenaline (#4) and kojic acid (#56) clusters in response to DMSO (D1 and D2).. 4

Supplementary Table S1: Summary of RNA-Seq data set and alignments statistic . …………5

Supplementary Table S2. GO terms significantly enriched in response to DMSO.Table S2.xlsx

Supplementary Table S3. Details of gene expression regulation of the 56 Secondary Metabolites Gene Clusters of *A. flavus* in response to DMSO (D1 and D2) ....Table S3.xlsx

Supplementary Table S4.. Normalized level of some *A. flavus* secondary metabolites detected in fungal and culture medium extracts ....Table S4.xlsx

Supplementary Table S5. Genes orthologous of *Aspergillus nidulans* genes determined as potential direct target gene for *VosA*. ....Table S5.xlsx

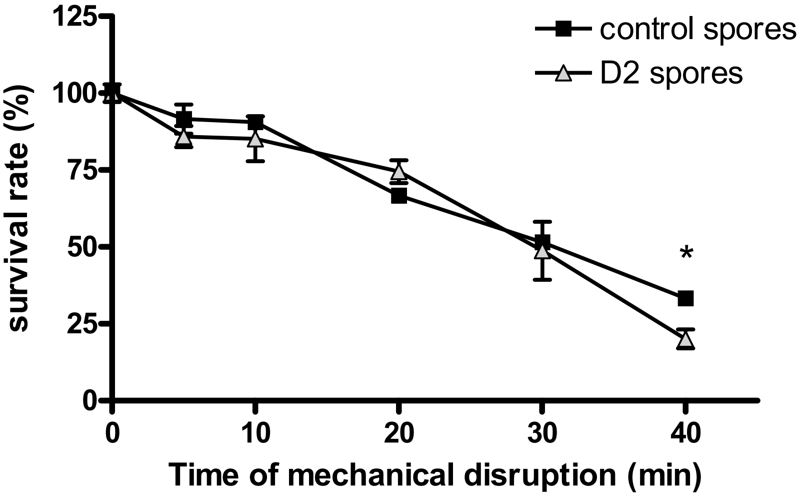

Figure S1*: Survival rate of pigmented (control) and non-pigmented (D2) conidia related to the duration of mechanical disruption by ceramic beads*. *No significant differences were observed after a Mann-Whitney test on final CFU obtained at each disruption time.*

A

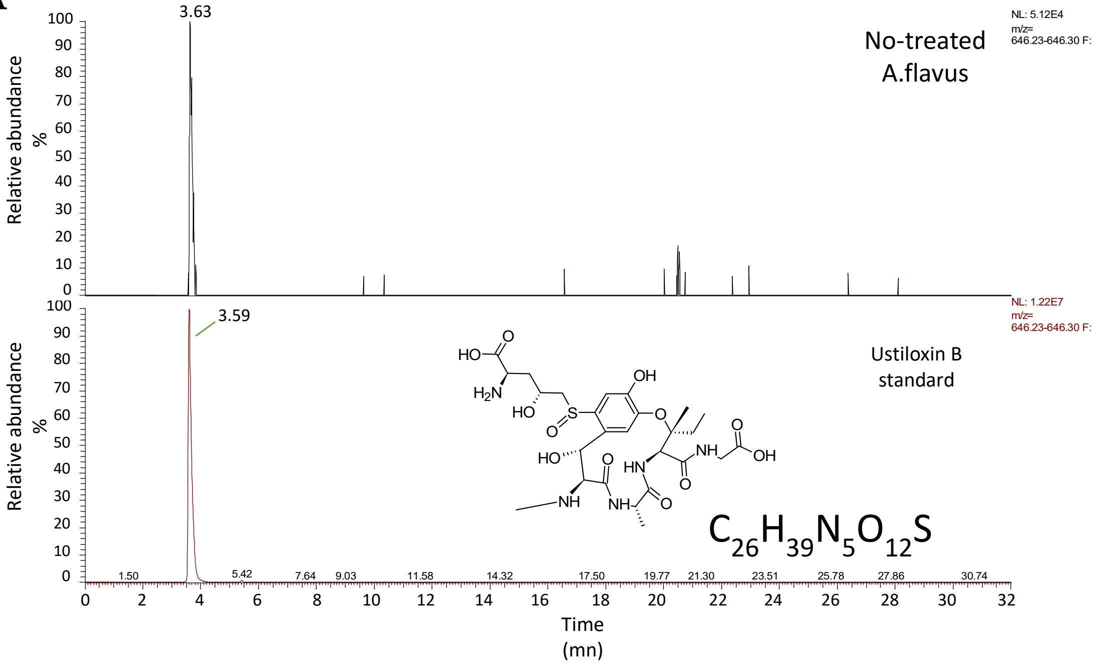

B

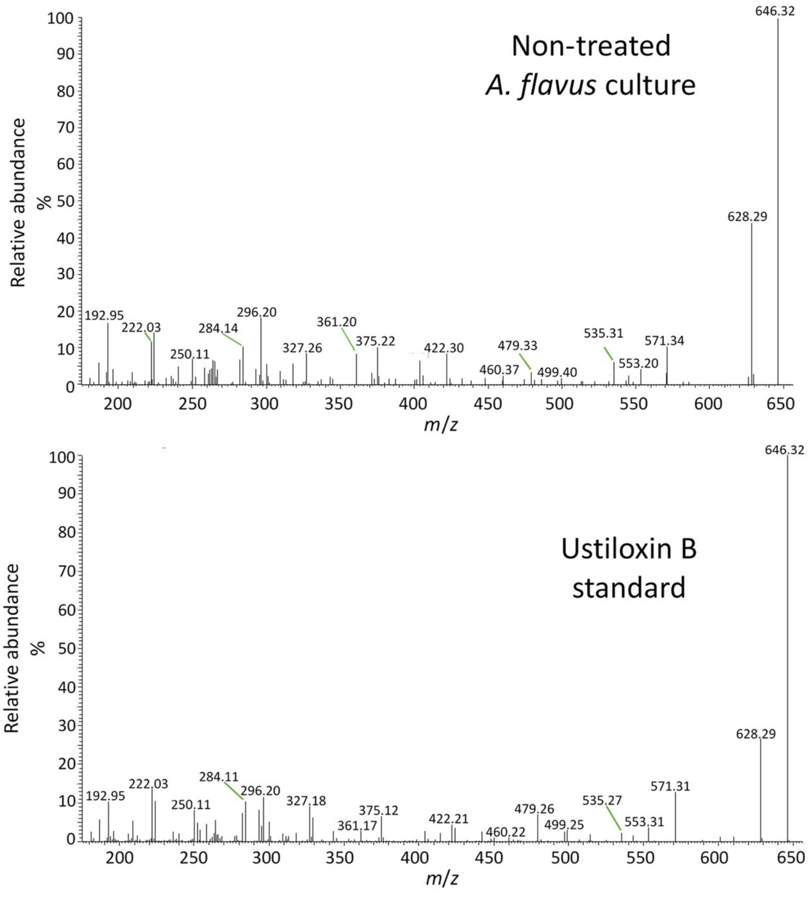

Figure S2: LC-MS chromatograms of non-treated *Aspergillus flavus* culture extracts and Ustiloxin B authentic standard (ESI+ mode), m/z corresponding in positive mode to ustiloxin B is 646.32. **B.** Comparison of positive mass fragmentation (MS/MS) of m/z 646.32 between the non-treated *A. flavus* culture extract and ustiloxin B standard.

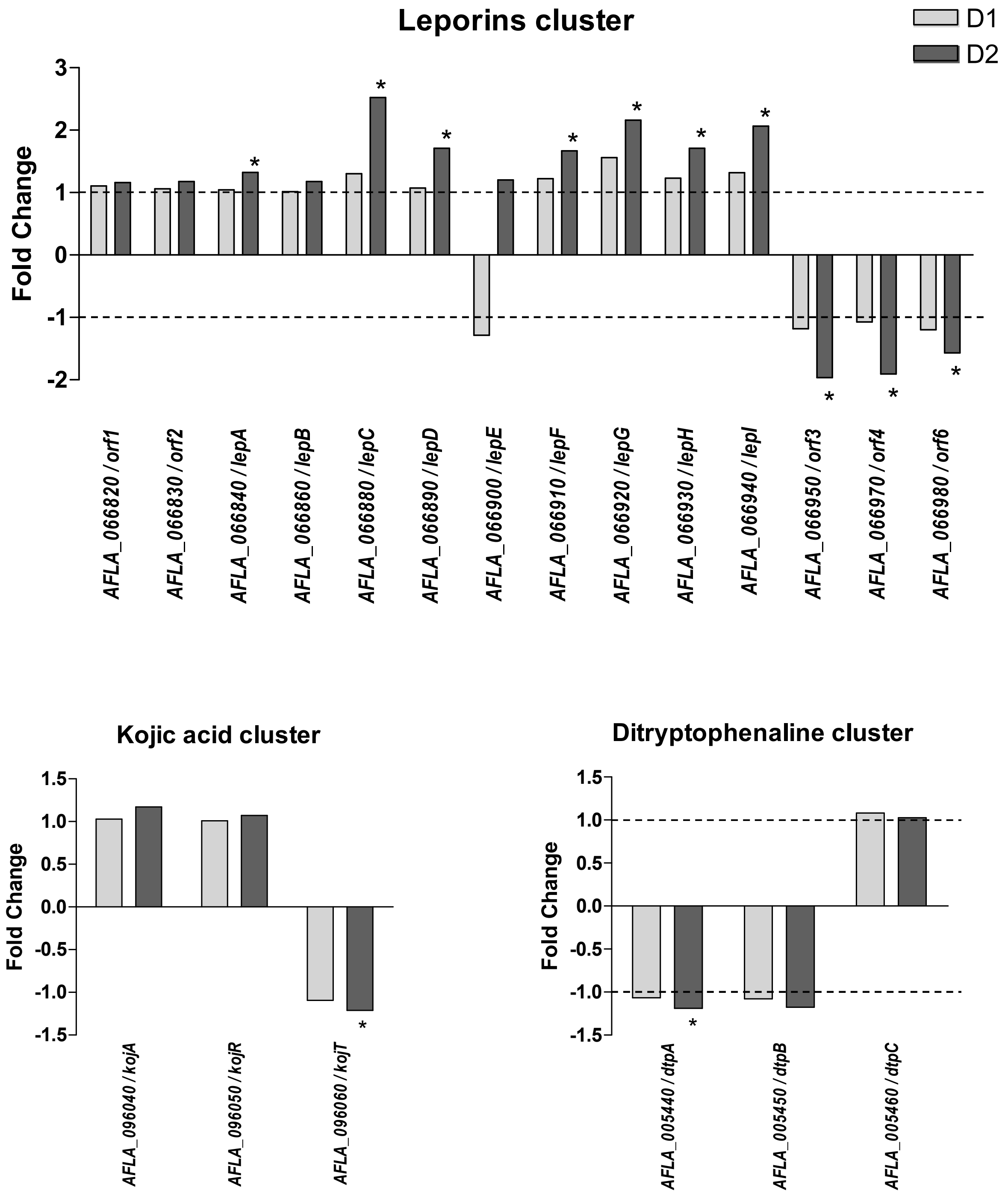

Figure S3: Fold Change expression of genes belonging to the mixed regulated leporins (#23), ditryptophenaline (#4) and kojic acid (#56) clusters in response to DMSO (D1 and D2). * q-value <0.05; dotted line represents abs(FC)=1 with no difference in expression against control samples

Table S1: Summary of RNA-Seq data set and alignments statistic

Samples	Raw reads	Clean reads	Average clean read length	% of uniquely mapped reads	% of reads mapped to multiple loci	% of unmapped reads	% of mapped reads	Nb of gene-assigned reads
T_1	25635990	25590686	297	73,48%	2,03%	24,48%	75,5%	14734082
T_2	22486877	22456674	297	73,30%	2,03%	24,67%	75,3%	12718457
T_3	23626049	23598390	297	78,96%	1,68%	19,36%	80,6%	15046553
T_4	18318728	18280062	296	77,14%	1,77%	21,09%	78,9%	11340720
T_5	14172452	14146604	295	73,84%	2,05%	24,11%	75,9%	8062727
T_6	22208354	22177148	296	76,11%	1,91%	21,98%	78,0%	13317145
D1_1	18373293	18343410	297	72,00%	2,15%	25,86%	74,1%	10137706
D1_2	22827805	22795332	297	72,71%	2,08%	25,21%	74,8%	12779417
D1_3	22216919	22187683	297	74,76%	1,94%	23,29%	76,7%	12985526
D1_4	22433855	22380103	293	72,99%	1,99%	25,01%	75,0%	12611554
D1_5	26444797	26412874	296	75,65%	1,87%	22,47%	77,5%	15452407
D1_6	23998067	23969648	294	71,81%	2,05%	26,14%	73,9%	12732164
D2_1	27612436	27555267	296	76,06%	1,77%	22,17%	77,8%	16648445
D2_2	21929663	21901732	297	79,60%	1,72%	18,67%	81,3%	13996278
D2_3	18826190	18799261	297	75,58%	1,86%	22,55%	77,5%	11143899
D2_4	21527734	21483452	297	74,65%	1,92%	23,42%	76,6%	12243644
D2_5	19172514	19139783	295	76,05%	1,88%	22,06%	77,9%	11266634
D2_6	21267233	21239400	294	77,20%	1,79%	21,00%	79,0%	12789021
**Mean**	**21837720**	**21803195**	**296 pb**	**75,11%**	**1,92%**	**22,97%**	**77,03%**	**12778132**

Table S5: genes orthologous of *Aspergillus nidulans* genes determined as potential direct target gene for VosA

Table S5: genes orthologous of *Aspergillus nidulans* genes determined as potential direct target gene for VosA (Wu et al, 2021)[a]

Aspergillus flavus NRRL 3357 strain Gene ID	*Aspergillus nidulans* FGSC A4 strain Gene ID	Putative function	Fold Change	Adjusted *p*-value
AFLA_049070	AN6642	sodium potassium-type ATPase putative *enaA*	4.03	4.93E-13
AFLA_003760	AN0391	NACHT domain protein.	4.03	6.64E-15
AFLA_138670	AN2822	aquaporin putative. Has domain(s) with predicted transporter activity, role in transport and membrane localization	4.13	4.27E-06
AFLA_048440	AN8829	conserved hypothetical protein	4.19	2.10E-13
AFLA_074270	AN4482	sugar transporter putative. Has domain(s) with predicted transmembrane transporter activity, role in transmembrane transport and integral component of membrane localization	4.19	5.58E-12
AFLA_044790	Con10 duplicated	conidiation-specific family protein	4.25	9.02E-11
AFLA_099050	AN10040	conserved hypothetical protein. Ortholog of A. fumigatus Af293 : Afu5g09180, A. niger CBS 513.88 : An07g03930, Penicillium expansum: Pexp_018490, P. rubens Pc20g10220	4.35	3.43E-15
AFLA_043550	AN1605	conserved hypothetical protein	4.80	1.36E-12
AFLA_055690	AN6581	ABC drug exporter AtrF	4.94	1.28E-14
AFLA_083110	AN5015	orthologous of *N. crassa* conidiation-specific protein Con10 (ConJ)	5.01	4.66E-13
AFLA_109380	AN0599	alcohol dehydrogenase putative	5.51	4.67E-12
AFLA_055700	AN6581	ATP-binding cassette transporter atrF	5.51	2.73E-14
AFLA_044800	AN8640	orthologous of *N. crassa* conidiation protein Con6 (ConF)	5.59	1.85E-13
AFLA_126170	AN2535	conserved hypothetical protein	6.09	7.52E-14

[a] Wu MY, Mead ME, Lee MK, Neuhaus GF, Adpressa DA, Martien JI, Son YE, Moon H, Amador-Noguez D, Han KH, Rokas A, Loesgen S, Yu JH, Park HS. 2021.
Transcriptomic, Protein-DNA Interaction, and Metabolomic Studies of VosA, VelB, and WetA in *Aspergillus nidulans* Asexual Spores. mBio. 12:e03128-20. doi: 10.1128/mBio.03128-20
